# PyHMMER: a Python library binding to HMMER for efficient sequence analysis

**DOI:** 10.1093/bioinformatics/btad214

**Published:** 2023-04-19

**Authors:** Martin Larralde, Georg Zeller

**Affiliations:** Structural and Computational Biology Unit, EMBL, Meyerhofstraße 1, Heidelberg 69117, Germany; Structural and Computational Biology Unit, EMBL, Meyerhofstraße 1, Heidelberg 69117, Germany

## Abstract

**Summary:**

PyHMMER provides Python integration of the popular profile Hidden Markov Model software HMMER via Cython bindings. This allows the annotation of protein sequences with profile HMMs and building new ones directly with Python. PyHMMER increases flexibility of use, allowing creating queries directly from Python code, launching searches, and obtaining results without I/O, or accessing previously unavailable statistics like uncorrected *P*-values. A new parallelization model greatly improves performance when running multithreaded searches, while producing the exact same results as HMMER.

**Availability and implementation:**

PyHMMER supports all modern Python versions (Python 3.6+) and similar platforms as HMMER (x86 or PowerPC UNIX systems). Pre-compiled packages are released via PyPI (https://pypi.org/project/pyhmmer/) and Bioconda (https://anaconda.org/bioconda/pyhmmer). The PyHMMER source code is available under the terms of the open-source MIT licence and hosted on GitHub (https://github.com/althonos/pyhmmer); its documentation is available on ReadTheDocs (https://pyhmmer.readthedocs.io).

## 1 Introduction

Protein similarity search and annotation is a key part of biological sequence analysis, allowing for the discovery of protein function from sequence data. Improving on Position-Specific Scoring Matrices that were used historically to search for motifs, Profile Hidden Markov Models (pHMMs) were introduced in HMMER (Eddy 2011) to model protein sequence families. Since then, HMMER has become a de facto standard for protein domain annotation, with several protein domain databases such as Pfam (Mistry et al. 2021) or TIGRFAM (Haft et al. 2013) being distributed as pHMMs.

HMMER provides different utilities with a command line interface (CLI), and only offers an application programming interface (API) for the C language (Kernighan and Ritchie 1978). This makes it rather cumbersome to use with modern languages such as Python, which are now more popular among scientists (Perkel 2015). Here we describe PyHMMER, a library providing Python bindings to the C API of HMMER using Cython (Behnel et al. 2011), allowing seamless integration of HMMER in larger Python programs, or in Jupyter notebooks (Kluyver et al. 2016). Such literate programming efforts help to address the reproducibility crisis, allowing for exact versioning and reproducible scripts for pHMM generation, among other recommended practices (Rule et al. 2019).

## 2 Implementation

The original HMMER codebase is organized into several components: a general purpose library for biological sequence manipulation named easel, a core library specific to HMMER named libhmmer, and dedicated CLI tools for building HMMs and performing sequence searches (hmmbuild, hmmsearch, phmmer, etc.).

The Cython language, a superset of Python that can be compiled into C or C++ extension modules, allows defining foreign function interfaces to the library components of HMMER. Using this particular feature, we developed the pyhmmer.easel and pyhmmer.plan7 extension modules to wrap the most relevant types from the easel and libhmmer libraries, respectively. In both submodules these types are exposed as Python classes, allowing the user to create and manipulate a Sequence or an HMM object directly (Listing 1).

Classes in PyHMMER implement the relevant methods from the Python data model: containers like TopHits or Domains support the usual Python syntax for iterating or indexing, while file readers support the context manager protocol to be used inside a with statement and the iteration protocol to read the file content with a simple for loop. Subclassing is supported to allow end-users to implement additional functionalities if needed. Numeric collections, such as VectorF which wraps a one-dimensional array of floating point numbers, all implement the buffer protocol. They can be used seamlessly with the entire NumPy ecosystem (Harris et al. 2020), using the numpy.asarray function to wrap them into an ndarray without copying the underlying data. Type hints are provided for all public classes and functions, allowing a static analyzer such as MyPy (https://mypy-lang.org) to detect type errors ahead of runtime. These type annotations also make PyHMMER more pleasant to use inside an Integrated Development Environment (IDE), where the function signatures can be suggested and corrected automatically.

For sensible types, the corresponding Python class also exposes internal attributes through Python properties that can be used for introspection. This includes some attributes that were not originally available in the HMMER results, such as the uncorrected p-value for each alignment (HMMER would only report the Bonferroni-corrected *P*-values in the output tables). HMM objects can be modified manually, for instance to set bitscore cutoffs using an externally computed threshold.

## 3 Results

The functionality of several of the CLI tools from HMMER was rewritten as pure Python functions using the API from the pyhmmer.easel and pyhmmer.plan7 modules.

**Figure 1:**
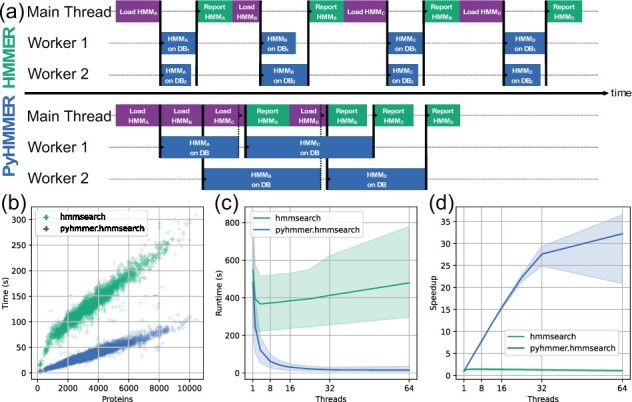
Comparison of the parallelization strategy for running *hmmsearch*. (a) A summary of the job dispatching between the HMMER and the PyHMMER implementations of *hmmsearch*. HMMER breaks the target database into chunks and synchronizes the queries; PyHMMER runs multiple queries in parallel threads while using the main thread for I/O. Blocking and non-blocking inter-thread communications are shown in plain and dashed lines, respectively. (b) Runtime comparison of *hmmsearch* annotation with six threads. Proteins were obtained from representative microbial genomes contained in the proGenomes2 database (Mende et al. 2020), and annotated with Pfam version 33.1 (Mistry et al. 2021) grouped by genome. Each command was run once on an Intel i7-10710U processor with six physical cores. (c) Average runtime and (d) speedup of *hmmsearch* implemented in PyHMMER compared to HMMER. Proteins of 10 different species were downloaded from proGenomes and annotated with Pfam version 35.0. Shown are the averages of three runs on an Intel Xeon E5-2683 processor with 16 physical cores. More benchmarks can be found at https://pyhmmer.readthedocs.io/en/latest/benchmarks.html.

Computational efficiency of the new parallelization code was benchmarked against the original implementation for the hmmsearch task (Fig. 1b–d). It shows much better performance for smaller target databases such as the proteome of a single microbial species, which can be entirely loaded into memory before querying. For extreme cases where none of the HMM or sequence databases fit into memory, a fallback using file readers is implemented to support larger searches at the cost of I/O overhead.

**Listing 1:**
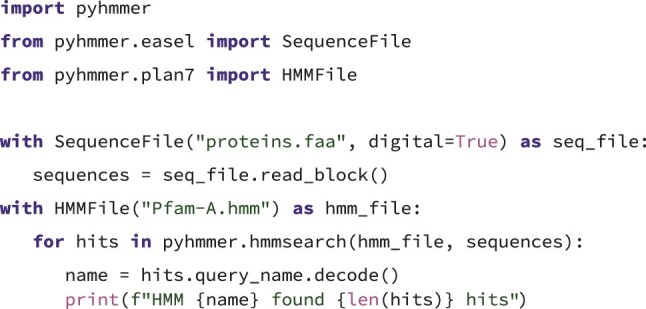
A Python code snippet demonstrating how to run *hmmsearch* with the PyHMMER API. The target sequences are pre-fetched from the proteins.faa file before running the search loop while the query HMMs are loaded iteratively from Pfam-A.hmm. More code examples can be found at https://pyhmmer.readthedocs.io/en/latest/examples/.

The parallel section in particular was reimplemented with plain Python threads and a different model for balancing load across worker threads (Fig. 1a), the default number of which is chosen as the number of physical cores to avoid resource contention. The hmmpgmdb client was adapted to use Python sockets for network communication, although message encoding and decoding uses the HMMER code for consistency.

The new threading strategy reduces latency caused by the filesystem as it introduces pre-fetching of query HMMs from the HMM file while the worker threads run the one-to-many comparison pipeline. Modeling the speedup with Amdahl’s law (Amdahl 1967) suggests that the original *hmmsearch* task is not taking full advantage of multi-core machines, with only around 35% of the code being truly parallel (Fig. 1d). In comparison, PyHMMER has ∼96% of the code in parallel sections. In practice, using PyHMMER to annotate a large protein set on a six-core machine reduced the runtime by 72%, totaling only 27 h where HMMER took 97 h (Fig. 1b). Similarly, *hmmscan* tasks benefit from the PyHMMER parallelization strategy, which makes it possible to annotate 1 million proteins with 32 threads within ∼2.5 h compared to an estimate of >7 days the original HMMER implementation would take.

At the time of writing, PyHMMER has already been integrated into several projects covering various areas of bioinformatics, including long-read transcriptome sequencing (Lienhard et al. 2021); average amino-acid identity estimation (Konstantinidis et al. 2022) or clustering of biosynthetic gene clusters (Kautsar et al. 2021).

## Data Availability

The data underlying this article are available on GitHub at https://github.com/althonos/pyhmmer. The datasets for benchmarking were derived from sources free for academic and non-commercial use: proGenomes 3 (https://progenomes.embl.de), Pfam 33.1 (https://www.ebi.ac.uk/interpro/).
